# FDG-PET in possible cardiac sarcoidosis: Right ventricular uptake and high total cardiac metabolic activity predict cardiovascular events

**DOI:** 10.1007/s12350-019-01659-2

**Published:** 2019-02-27

**Authors:** Heikki Tuominen, Atte Haarala, Antti Tikkakoski, Mika Kähönen, Kjell Nikus, Kalle Sipilä

**Affiliations:** 1 Department of Clinical Physiology and Nuclear Medicine, Tampere University Hospital, 33520 Tampere, Finland; 2 Faculty of Medicine and Health Technology, Tampere University, Tampere, Finland; 3 Department of Cardiology, Heart Center, Tampere University Hospital, Tampere, Finland

**Keywords:** Sarcoid heart dusease, inflammation, PET, metabolism imaging agents

## Abstract

**Background:**

Cardiac involvement accounts for the majority of morbidity and mortality in sarcoidosis. Pathological myocardial fluorodeoxyglucose (FDG)-uptake in positron emission tomography (PET) has been associated with cardiovascular events and quantitative metabolic parameters have been shown to add prognostic value. Our aim was to study whether the pattern of pathological cardiac FDG-uptake and quantitative parameters are able to predict cardiovascular events in patients with suspected cardiac sarcoidosis (CS).

**Methods:**

137 FDG-PET examinations performed in Tampere University Hospital were retrospectively analyzed visually and quantitatively. Location of pathological uptake was noted and pathological metabolic volume, average standardized uptake value (SUV), and total cardiac metabolic activity (tCMA) were calculated. Patients were followed for ventricular tachycardia, decrease in left ventricular ejection fraction, and death.

**Results:**

Eleven patients had one or more cardiovascular events during the follow-up. Five patients out of 12 with uptake in both ventricles had an event during follow-up. Eight patients had high tCMA (> 900 MBq) and three of them had a cardiovascular event. Right ventricular uptake and tCMA were significantly associated with cardiovascular events during follow-up (*P*-value .001 and .018, respectively).

**Conclusions:**

High tCMA and right ventricular uptake were significant risk markers for cardiac events among patient with suspected CS.

**Electronic supplementary material:**

The online version of this article (10.1007/s12350-019-01659-2) contains supplementary material, which is available to authorized users.

## Introduction

Detecting cardiac involvement in sarcoidosis is essential, as it accounts for the majority of sarcoidosis-related deaths.^1^–^3^ Cardiac ^18^fluorodeoxyglucose positron emission tomography/computerized tomography (18FDG PET/CT) (later referred to as PET) has been used to identify active cardiac inflammation with a sensitivity and specificity of 89% and 78%, respectively.^4^

In early studies, the prognosis of cardiac sarcoidosis (CS) was poor and resulted in death within 2 years.^5^ Later studies showed an overall improvement of prognosis. In a nationwide Finnish study of 110 CS patients, 9% died during a median follow-up of 79 months.^6^ Left ventricular ejection fraction (LVEF) was the most powerful prognostic factor in CS patients treated with corticosteroids.^7^ It is essential to start corticosteroid treatment early, as recovery of left ventricular function is more pronounced in patients with only moderately decreased LVEF. In patients with LVEF < 30%, corticosteroid therapy did not improve cardiac function.^8^ In addition to degrading function, CS may also cause ventricular arrhythmias, sometimes with fatal consequences.^9^–^11^

Existing literature on the prognostic value of PET is relatively scarce, but it has been shown to enhance the prognostic assessment of CS beyond the Japanese Ministry of Health and Welfare (JMWH) criteria.^12^,^13^ According to Ahmadian et al., total cardiac metabolic activity (tCMA) independently predicted cardiac events in patients with previously diagnosed sarcoidosis.^14^ On the other hand, a study comparing findings in consecutive PET- and cardiac magnetic resonance imaging (CMR) studies reported no prognostic value of PET-findings among patients with late gadolinium enhancement (LGE) in CMR.^15^

Our aim was to study how visual and quantitative PET-imaging parameters performed in predicting cardiovascular (CV) events in a clinical cohort of patients referred to PET-imaging for suspected CS.

## Methods

### Study Population

We retrospectively screened all cardiac PET studies performed in the Tampere University Hospital from August 2012 to September 2015. We excluded studies, in which the clinical indication was not CS suspicion, if the patient’s clinical data could not be obtained or PET-study was performed for follow-up of previously diagnosed CS. Imaging studies were also excluded if the dedicated imaging protocol described below was not followed precisely. Altogether, 137 PET examinations were analyzed for the present study. In our hospital, PET is routinely performed in the diagnostic workup of possible CS. The reason for a clinical suspicion of CS was one or more of the following: unexplained atrio-ventricular (AV) block (*n* = 61), ventricular (*n* = 39), or supraventricular (*n* = 13) arrhythmia, unexplained dilated cardiomyopathy (*n* = 27), or unexplained low ejection fraction on echocardiography (*n* = 46), other echocardiographic findings suggestive of CS (*n* = 53), or syncope (*n* = 27). The referring cardiologist considered the symptoms and clinical findings as inconclusive after routine evaluation, including clinical examination, ECG, and echocardiography. Coronary angiography was performed in cases with a clinical suspicion of coronary artery disease.

### PET-Imaging

All patients underwent an integrated PET/CT (Discovery STE 16, GE Healthcare, Milwaukee, WI, USA) examination. To minimize physiological myocardial FDG-uptake, the patients were instructed not to consume any carbohydrates during the day before the imaging exam and to fast for 12 h before the FDG injection. The patients maintained a food diary during the prescribed diet. The patients were also instructed to avoid heavy physical exercise to minimize FDG-uptake in skeletal muscle. Patient height and weight were measured before the administration of the radiopharmaceutical, and blood glucose levels were tested to confirm a level of < 7 mmol/l. The PET/CT images were acquired approximately 60 min after the intravenous injection of FDG using the Medrad^®^ Intego PET infusion system (Bayer Medical Care Inc., Indianola, PA, USA). In our hospital, the activity injected is based on the patient’s weight (3-3.2 MBq/kg). The imaging protocol has been described in more detail previously.^16^

### Analysis of PET Images

We classified the uptake pattern in the left ventricular (LV) myocardium according to the recommendations of the Japanese Society of Nuclear Cardiology as ‘none’ (no activity exceeding normal blood pool activity), ‘global diffuse’ (uniform activity over the entire myocardium), ‘focal’ (focally increased spot(s) of activity, other regions inactive), ‘focal on diffuse’ (intense focal spot(s) of activity overlapping global myocardial activity), or ‘diffuse non-global’ (faint activity on at least two LV walls, but at least some areas of myocardium with no activity over normal blood pool activity). Diffuse non-global uptake is not mentioned in the Japanese Society of Nuclear Cardiology recommendations but was considered to be a similar physiological phenomenon as diffuse uptake. The uptake was considered physiological if it was classified as none, global diffuse, or diffuse non-global. Uptake was considered pathological if it was classified as focal or focal on diffuse. We also classified the uptake in the right ventricle (RV) in a similar manner. The maximum standardized uptake value (SUVmax) in the heart was measured and its location was determined. Extracardiac uptake in the lymph nodes was considered pathological if it exceeded that of the mediastinal blood pool. The areas inspected for possible pathological extracardiac uptake included lymph nodes in axillary, subclavicular, mediastinal, hilar, and epigastric sites. Pathological FDG-uptake in the lung parenchyma, liver, spleen, and bone marrow were also noted. Uptake in the liver, spleen and lung parenchyma was considered pathological if there were spots of metabolic activity exceeding the physiological uptake of the surrounding parenchyma. Bone marrow uptake was considered pathological if focal uptake exceeding that of the liver was noted. The images were interpreted after patient anonymization and randomization. The images were separately interpreted by two experienced nuclear medicine physicians (HT, 10 years of experience and KS, 15 years of experience) blinded to all clinical data. In cases where the interpretation of LV-uptake differed between the observers, a consensus was reached and used in further analyses. After this initial analysis, the quantitative parameters were measured using PETVCAR software on GE Advantage workstation (GE Healthcare, Milwaukee, WI, USA). Blood pool SUVmean was first measured from ascending aorta. A threshold for abnormal cardiac uptake was defined as blood pool SUVmean × 1.5. This value was used to delineate metabolic volume. SUVmean over metabolic volume was measured and used to calculate tCMA by multiplying the metabolic volume by SUVmean.^14^ Cut-off for high tCMA was defined as 900 MBq that is approximately tCMA + 2SD in the group with no events.

### Collection of Clinical Data

Clinical data were retrospectively collected from the electronic medical record system of Tampere University Hospital, which contains information from 2008 onward. We collected demographic information, echocardiography findings, MRI-findings, relevant diagnoses, symptoms and endomyocardial biopsy (EMB) findings, deaths, healthcare visits, and hospitalizations for ventricular tachyarrhythmia. Systolic function evaluated by echocardiography was categorized as normal (> 50%), decreased (35-50%) or (poor < 35%). Echocardiography findings were collected from studies performed nearest to PET-study and the last echocardiography study of the follow-up period. Diagnosis of CS by cardiologists was based on EMB or a combination of other biopsy verification of sarcoidosis and clinical and/or imaging findings indicating cardiac involvement. Electronic health record data for follow-up were available from the Tampere University Hospital.

### Cardiovascular Events

Cardiac events of interest were reduction in LVEF, hospitalization due to cardiac arrhythmia, and death. Change in LVEF was determined by comparing the findings between echocardiography studies performed nearest to the PET-study and the last study of the follow-up period. Decrease in LVEF was defined as a negative change in LVEF category defined above. We used hospital admission as a definition of severe ventricular arrhythmia as the number of other detected ventricular arrhythmias was highly dependent on whether or not the patient had a pacemaker or implanted cardioverter-defibrillator (ICD).

### Statistical Methods

Statistical analyses were performed using IBM SPSS statistics version 22.0 (Armonk, NY, USA) and R software version 3.2.2. *T* test was used to compare quantitative metabolic parameters between patients, who had CV events and those with no events. Comparison of quantitative parameters also included patients with physiological FDG-uptake. Chi square test was used to test the statistical difference in dichotomous variables between groups. Survival analysis was performed with log-rank test.

## Results

Baseline characteristics, PET-findings and treatment during follow-up for patients with and without CV events are presented in Table 1. 35 (26%) patients had a diagnosed sarcoidosis prior to imaging or during follow-up. Cardiac sarcoidosis was diagnosed in 18 (13%) patients and in 7 patients (5%) it was confirmed by EMB.

**Table 1:** Baseline characteristics, PET-findings and treatment during follow-up in patients with and without events during follow-up

	Patients with events *n* = 11	Patients with no events *n* = 126	*P*-value
Baseline characteristics			
Female sex	45% (5)	43% (54)	.556
Age, years (SD)	37 (8)	45 (13)	.062
Follow-up, mo (SD)	50 (8)	44 (11)	.059
Reduced LVEF at baseline	73% (8)	54% (68)	.190
PET			
Pathological LV-uptake	46% (5)	22% (28)	.091
Pathological RV-uptake	46% (5)	6% (7)	< .001
tCMA > 900 MBq	27% (3)	4% (5)	.018
Extracardiac uptake	36% (4)	20% (25)	.180
SUVmax	7.1 (5.6)	4.4 (2.8)	< .001
Metabolic volume (cm3)	105 (167)	44 (96)	.050
tCMA (MBq)	530 (886)	163 (388)	< .001
Treatment			
ICD	100% (11)	18% (12)	< .001
Anti-inflammatory medication	46% (5)	18% (22)	.041
CS-diagnosis by the end of follow-up	27% (3)	20% (15)	.148

Values are % (*n*) for dichotomous and mean (SD) for continuous variables

*LVEF*, left ventricular ejection fraction; *LV*, left ventricle; *RV*, right ventricle, *SUV*, standardized uptake value; metabolic volume, volume of myocardium with SUV > 1.5 × aortic reference SUV; *SUVmean*, mean SUV over pathological metabolic volume; *tCMA*, total cardiac metabolic activity calculated as SUVmean × metabolic volume; *ICD*, implantable cardioverter-defibrillator; *CS*, cardiac sarcoidosis

33 (24%) patients had pathological myocardial FDG-uptake: 28 patients had a focal and 5 had a focal on diffuse pattern. 12 patients (9%) had pathological FDG-uptake in RV. All the patients with RV-uptake also had LV-uptake. Of the 104 (76%) patients without pathological uptake 85 (82%) had no visible uptake, three (3%) had diffuse uptake over LV and 16 (15%) had diffuse non-global uptake pattern. One patient had diffuse and one had diffuse non-global uptake in RV myocardium.

The follow-up time varied between 25.2 to 62.6 months (2.1-5.2 year), mean 54.7 months (3.74 years). Altogether, 11 patients had at least one event during follow-up: seven were hospitalized because of ventricular tachycardia, five had worsening of systolic LV function and three patients died. There was no significant difference in baseline characteristics between patients, who had events, and those who did not. Also, cardiac events were not significantly more common in patients who were diagnosed with CS according to diagnostic criteria.

CMR had been performed in 37 patients, of whom 19 (51%) had LGE. Of those 19 patients four (21%) had a CV event during follow-up. In the subset of patients with CMR data, all those subjects who had a CV event also had LGE.

**Figure 1:**
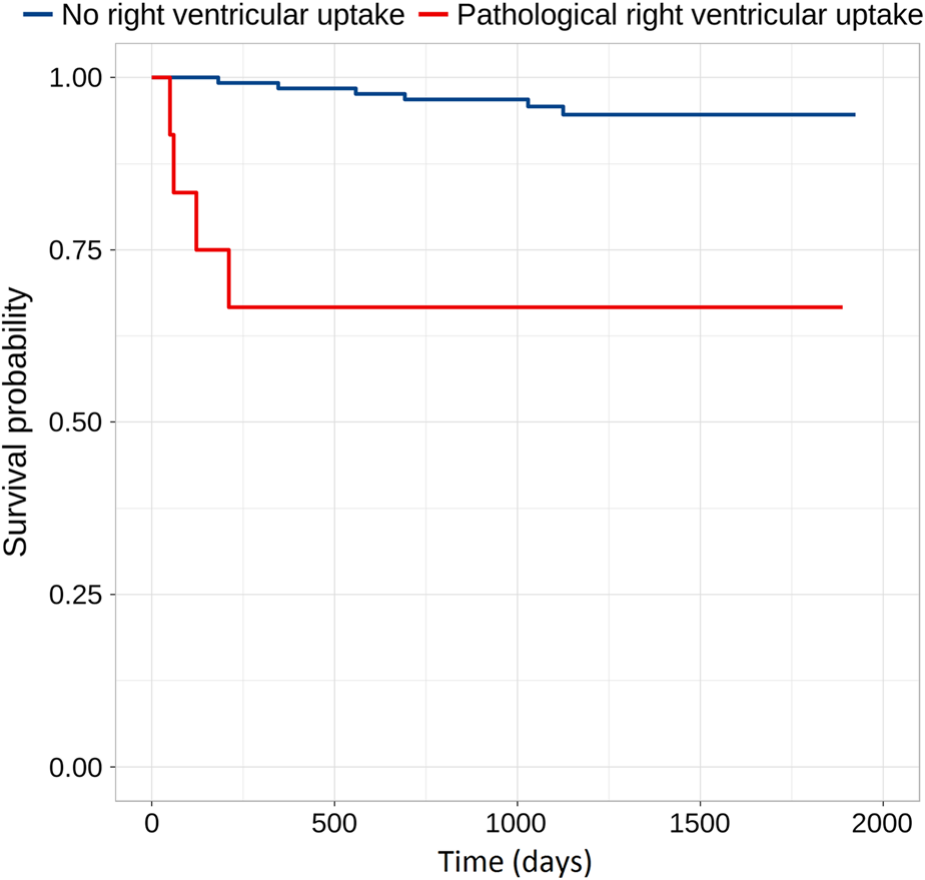
Survival free of cardiac events in patients with no right ventricular uptake and in those with pathological right ventricular uptake. Between groups the *P*-value is .001

Cardiac SUVmax and tCMA were higher in patients who had CV events compared to those with no events: 7.1 vs 4.4 and 530 MBq vs 163 MBq, respectively (*P* < .001 for both). There were 33 patients with abnormal LV-uptake on PET and 12 of them had abnormal uptake also in the RV. Five (15%) out of 33 patients with pathological cardiac uptake on PET had a CV event on follow-up. All five had pathological uptake also in the RV. Pathological RV-uptake was significantly more common in patients with CV events compared to those without events (*P* = .001). Event-free survival curves in patients with and without pathological RV-uptake are shown in Figure 1.

**Figure 2:**
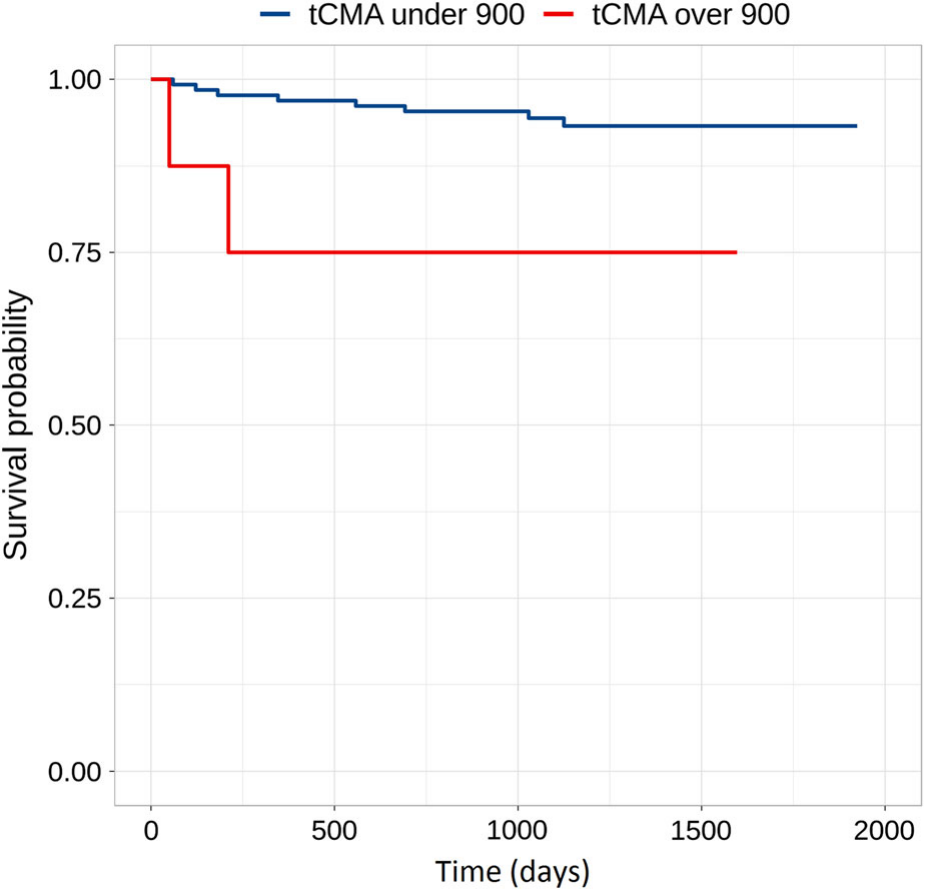
Survival free of cardiac events in patients with tCMA below or above 900 MBq. Between groups the *P*-value is .032

Eight patients had tCMA > 900 MBq and three (38%) of them had a CV event compared to only 6% of those with tCMA < 900 MBq. All these three patients also had pathological RV-uptake. Patients with pathological RV-uptake had significantly higher tCMA compared to those without RV-uptake: 914 (SD.706) vs. 123 (SD 356) MBq (*P* < .001). There were only 5 patients who had both high tCMA and RV-uptake, and 3 (60%) of them had a CV event. Event-free survival curves in patients with high and low tCMA are shown in Figure 2.

In survival analysis, pathological RV-uptake was a significant predictor of cardiac events (*P* = .001). Also high tCMA > 900 MBq significantly predicted events (*P* = .032). Decreased baseline LVEF, pathological LV-uptake or extracardiac FDG-uptake did not significantly predict CV events during follow-up (*P* = .094, .217 and .489, respectively).

## Discussion

We examined the value of cardiac FDG-PET in predicting CV events in patients with a suspicion of CS. Pathological RV-uptake was the strongest imaging-related predictor of future CV events defined as a decrease in LVEF, hospitalization for ventricular tachycardia (VT), or death. Also, the quantitative parameter tCMA was a significant predictor of events.

Decreased LVEF has been recognized as an important predictor of long-term outcome in CS patients treated with corticosteroids.^7^ In our population, the majority of the patients who suffered for cardiac events had decreased LVEF at baseline. However, decreased LVEF had poor predictive value, as the majority of the patients in our population were referred for imaging because of unexplained cardiac failure. In fact, pathological cardiac FDG-uptake was more common among patients with normal LVEF that in those with cardiac failure.^16^ This reflects the problem of recognizing the patients with cardiac findings with increased risk for major cardiac events.

Previously Blankstein et al. have reported that pathological cardiac PET-findings predict VT and mortality better than a CS-diagnosis by JMWH criteria and independently of LVEF.^12^ FDG-uptake in the RV indicated a fivefold risk of cardiac events compared to those with normal PET and myocardial perfusion in their study. In another study, myocardial perfusion-metabolism mismatch (metabolism assessed by PET) was related to cardiac events in patients assessed for possible cardiac sarcoidosis.^13^ PET has also been shown to predict major cardiac events in patients with unexplained atrio-ventricular block, a common manifestation of sarcoidosis.^17^

Furthermore, quantitative evaluation of myocardial metabolic activity in PET has been shown to predict cardiac events in some studies. Muser et al. showed that a temporal decrease in tCMA indicated a lower risk of events.^18^ Ishiyama et al. reported that SUVmax over all sarcoidosis-involved organs better predicted response to oral corticosteroids than tCMA in patients with CS. 19

LGE in CMR has been shown to be a strong predictor of cardiac events in patients with proven systemic sarcoidosis even in patients with preserved LVEF.^20^,^21^ Bravo et al. studied PET and CMR in patients with clinical manifestations of CS and found that LGE predicted cardiac events similarly in PET-positive and PET-negative patients. They concluded that the risk of sustained VT and death were associated with myocardial scarring but not to active inflammation.^15^ However, they did not analyze PET-findings beyond negative/positive. Indeed, PET positivity per se was not predictive of cardiac events in our population either. The predictive value in our study was particularly associated with pathological RV-uptake. Interestingly, Murtagh et al. showed that ventricular dysfunction in addition to the large burden of cardiac LGE denoted a high risk of cardiac events.^21^ In another recent study, it was shown that RV LGE predicted cardiac events.^22^ Both MRI and PET studies confirm the prognostic importance of abnormal RV imaging findings. Moreover, electrophysiological studies have shown that RV scarring is a common finding in CS patients with VT.^23^ RV scarring was often found to be confluent in comparison to patchy scarring in the left ventricle, providing large amount of substrate for re-entry circuits.^24^

In our population CMR was performed only in a subset of 39 patients of whom 19 (51%) had LGE. There was an association between reported LGE and CV events (*P*-value .039). Significantly, in this subset of available CMR data, there were no CV events in patients without LGE. Thus, negative CMR seems to have very good negative predictive value. As the number of events was low we could not perform further analyses for independent role of PET-findings in this subgroup. However, a significant proportion of patients suspected for CS had a pacemaker or ICD, and in those patients CMR may not be feasible and for this reason we consider our results on the prognostic role of PET-findings relevant.

We also found that the quantitative parameter tCMA predicts cardiac events. This is in line with the results reported by Ahmadian et al., that high tCMA predicts ventricular tachyarrhythmias and death.^14^ However, they did not report the possible pathological uptake in RV. Sixty-three percent of our 8 patients in high tCMA group had RV-uptake, and all the high tCMA patients who had events also had pathological RV-uptake. The combination of RV-uptake and high tCMA signified 60% risk for events and should always be communicated clearly to the clinician. Right ventricular uptake is easily recognized while interpreting PET studies making it a useful sign of high risk in every day practice.

In our population, only a minority (13%) of patients had sarcoidosis confirmed by biopsy. Other diseases like viral or giant cell myocarditis can cause pathological myocardial uptake. Interestingly, patients with diagnosed CS did not have more cardiac events than those without CS-diagnosis (Table 1). We consider this finding to be clinically significant as cardiologists should consider patients with a high burden of cardiac glycolysis and/or right ventricular RV-uptake to be a high-risk group whether or not they fulfill the diagnostic criteria for CS.

We had access only to the information in the patient record of Tampere University Hospital. Possible contacts to other health care facilities were not recorded and some endpoint events may have been missed. The number of recorded cardiac events during follow-up was relatively low. Due to the small number of events, multivariable analysis was not feasible. Also, CMR data were available only in a small proportion of the patients. This prevented us from analyzing the effect of myocardial scarring on the predictive value of PET-findings. This, however, is often the clinical reality in patients with a suspicion of CS due to conduction disturbances or VT requiring a pacemaker or ICD. Our population is heterogeneous and majority of the patients do not fulfill diagnostic criteria for CS. However, the population is representative of the true clinical situation and the results can be applied to everyday clinical decision making. Regrettably, we have no data on follow-up PET studies. Changes in tCMA and RV-uptake status by treatment and impact of these changes on prognosis will be an interesting topic for future research. Our population consists of white Caucasians and, as sarcoidosis is known to have different representations in different populations our results may not be generalizable to other populations.

## Conclusion

Among patients referred for FDG-PET imaging to assess possible cardiac sarcoidosis, pathological right ventricular uptake and a high burden of cardiac glycolysis predict adverse cardiac events during follow-up. A combination of pathological RV-uptake and high tCMA signified very high risk for future CV events. Nuclear medicine physicians should be aware of this and communicate such findings clearly to the clinician.

## New Knowledge Gained

Pathological FDG-uptake in right ventricle is easy to observe and should be considered as a significant risk marker in patients with suspected cardiac sarcoidosis especially if present in combination with high total cardiac metabolic activity.

## Electronic supplementary material

Below is the link to the electronic supplementary material.

Supplementary material 1 (PPTX 247 kb)

## Abbreviations

PET: Positron emission tomography
FDG: Fluorodeoxyglucose
CS: Cardiac sarcoidosis
LV: Left ventricle
RV: Right ventricle
CV: Cardiovascular
LVEF: Left ventricular ejection fraction
tCMA: Total cardiac metabolic activity
SUV: Standardized uptake value
AV: Atrio-ventricular
