# Supplementary material for: FDG-PET in possible cardiac sarcoidosis: Right ventricular uptake and high total cardiac metabolic activity predict cardiovascular events
Source: J Nucl Cardiol. 2019 Feb 27;28(1):199–205. doi: 10.1007/s12350-019-01659-2 (PMC7920884; doi:10.1007/s12350-019-01659-2)
Supplement: Supplementary file 1 — Supplementary material 1 (PPTX 247 kb) [file 12350_2019_1659_MOESM1_ESM.pptx]

## Slide 1
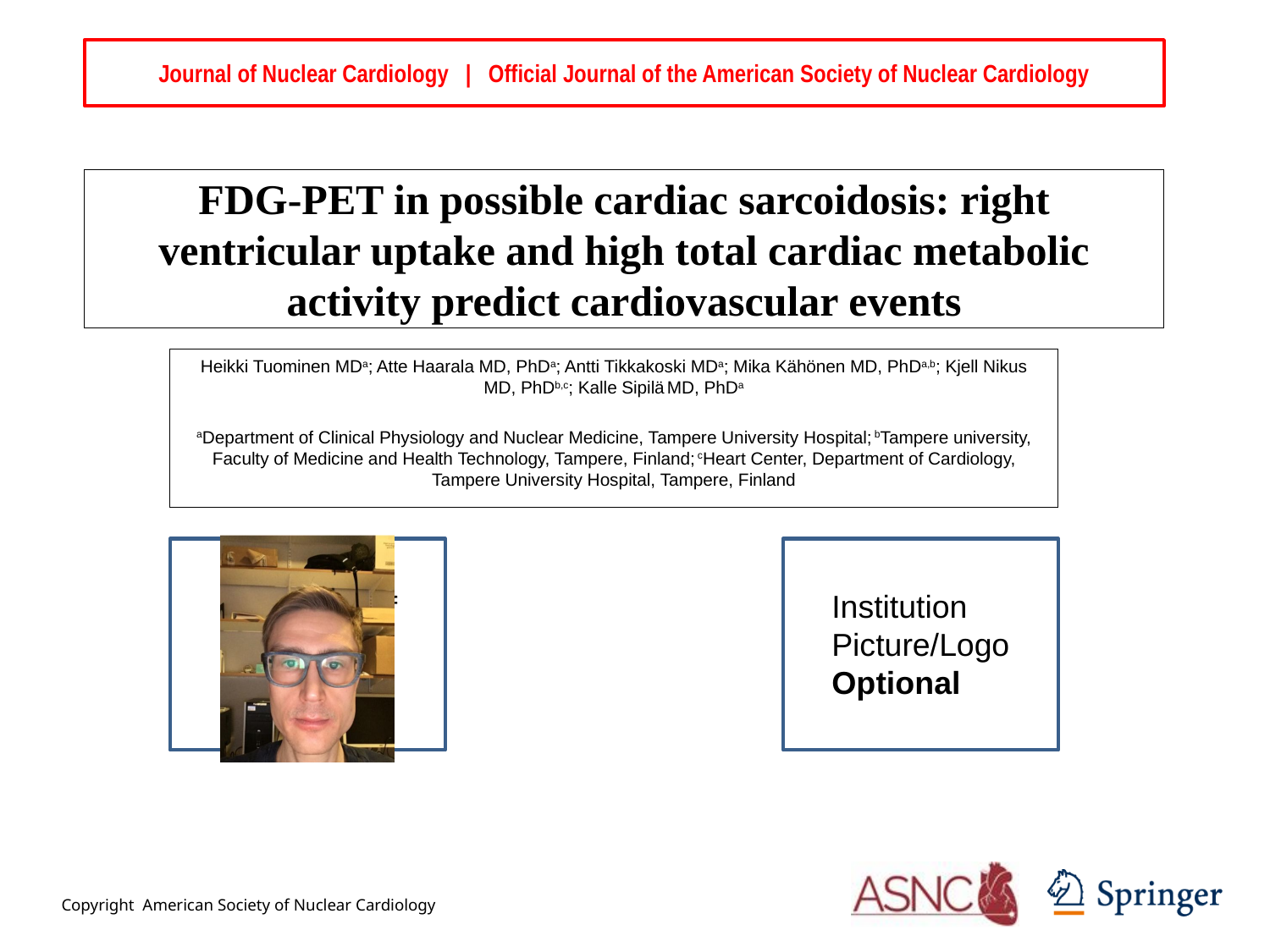

Journal of Nuclear Cardiology | Official Journal of the American Society of Nuclear Cardiology
# FDG-PET in possible cardiac sarcoidosis: right ventricular uptake and high total cardiac metabolic activity predict cardiovascular events
Heikki Tuominen MDa; Atte Haarala MD, PhDa; Antti Tikkakoski MDa; Mika Kähönen MD, PhDa,b; Kjell Nikus MD, PhDb,c; Kalle Sipilä MD, PhDa
aDepartment of Clinical Physiology and Nuclear Medicine, Tampere University Hospital; bTampere university, Faculty of Medicine and Health Technology, Tampere, Finland; cHeart Center, Department of Cardiology, Tampere University Hospital, Tampere, Finland
Head shot of author
required
Institution
Picture/Logo
Optional
Copyright American Society of Nuclear Cardiology

## Slide 2
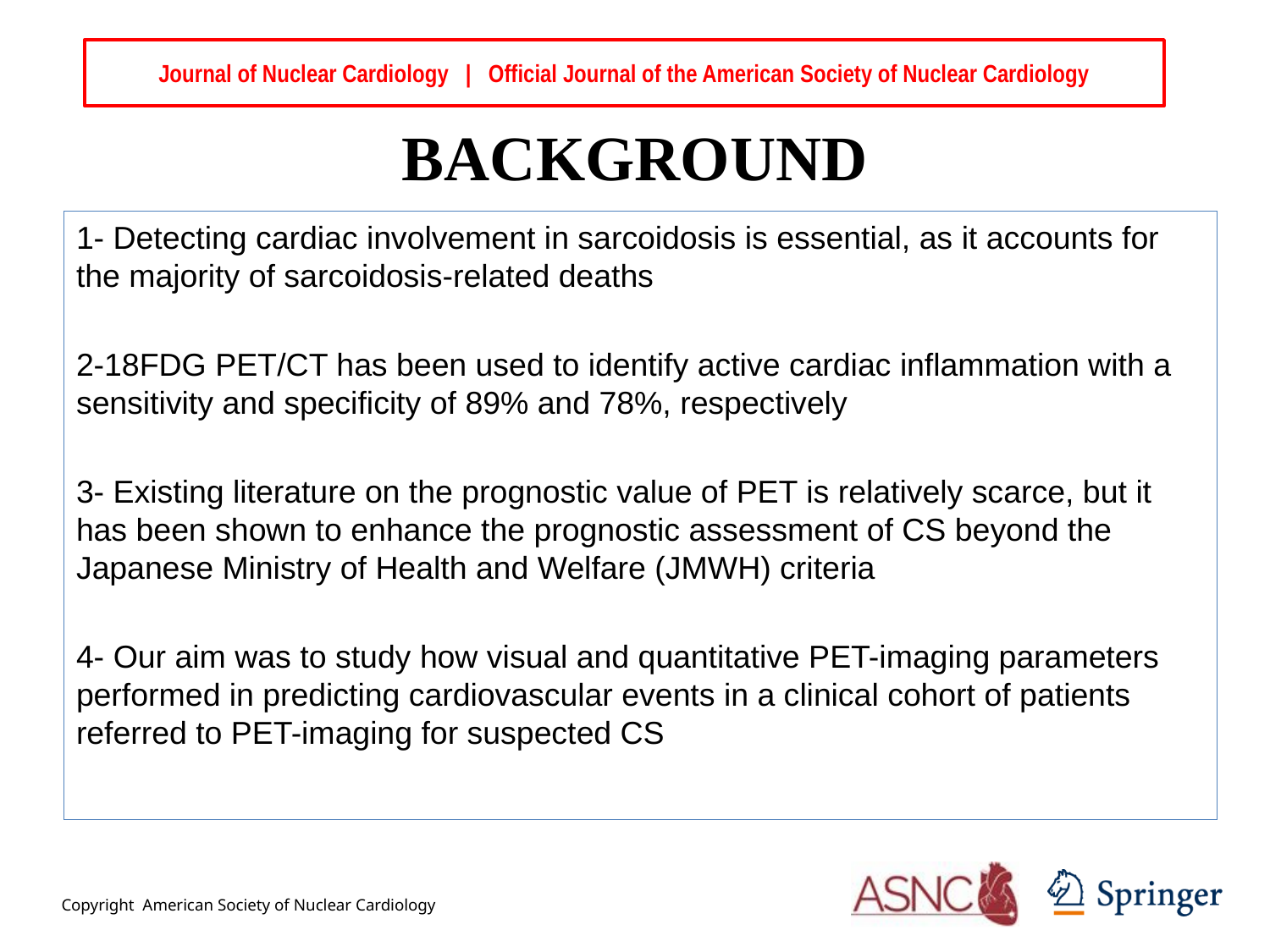

Journal of Nuclear Cardiology | Official Journal of the American Society of Nuclear Cardiology
# BACKGROUND
1- Detecting cardiac involvement in sarcoidosis is essential, as it accounts for the majority of sarcoidosis-related deaths
2-18FDG PET/CT has been used to identify active cardiac inflammation with a sensitivity and specificity of 89% and 78%, respectively
3- Existing literature on the prognostic value of PET is relatively scarce, but it has been shown to enhance the prognostic assessment of CS beyond the Japanese Ministry of Health and Welfare (JMWH) criteria
4- Our aim was to study how visual and quantitative PET-imaging parameters performed in predicting cardiovascular events in a clinical cohort of patients referred to PET-imaging for suspected CS
Copyright American Society of Nuclear Cardiology

## Slide 3
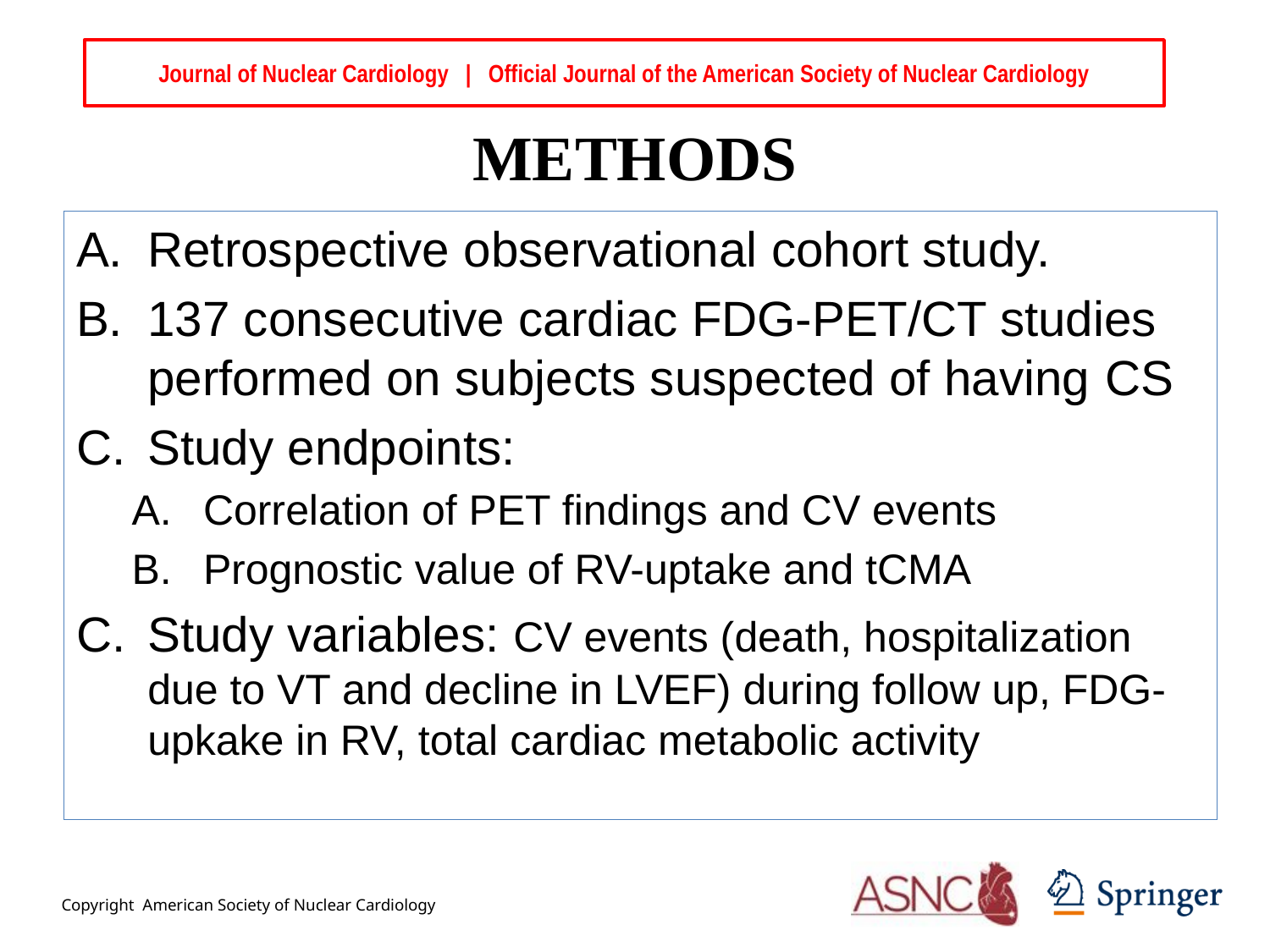

Journal of Nuclear Cardiology | Official Journal of the American Society of Nuclear Cardiology
# METHODS
Retrospective observational cohort study.
137 consecutive cardiac FDG-PET/CT studies performed on subjects suspected of having CS
Study endpoints:
Correlation of PET findings and CV events
Prognostic value of RV-uptake and tCMA
Study variables: CV events (death, hospitalization due to VT and decline in LVEF) during follow up, FDG-upkake in RV, total cardiac metabolic activity
Copyright American Society of Nuclear Cardiology

## Slide 4
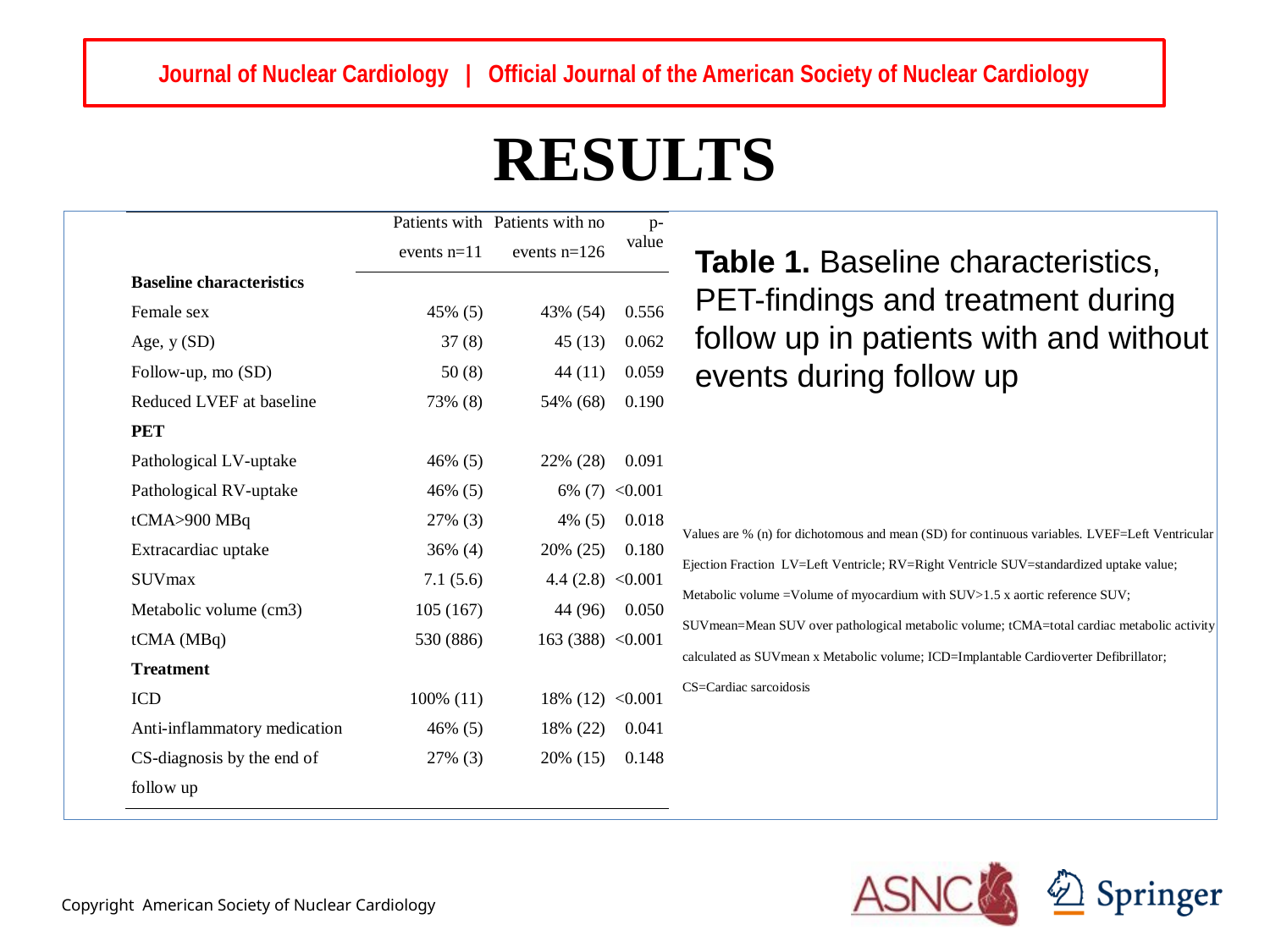

Journal of Nuclear Cardiology | Official Journal of the American Society of Nuclear Cardiology
# RESULTS
Table 1. Baseline characteristics, PET-findings and treatment during follow up in patients with and without events during follow up
Copyright American Society of Nuclear Cardiology

## Slide 5
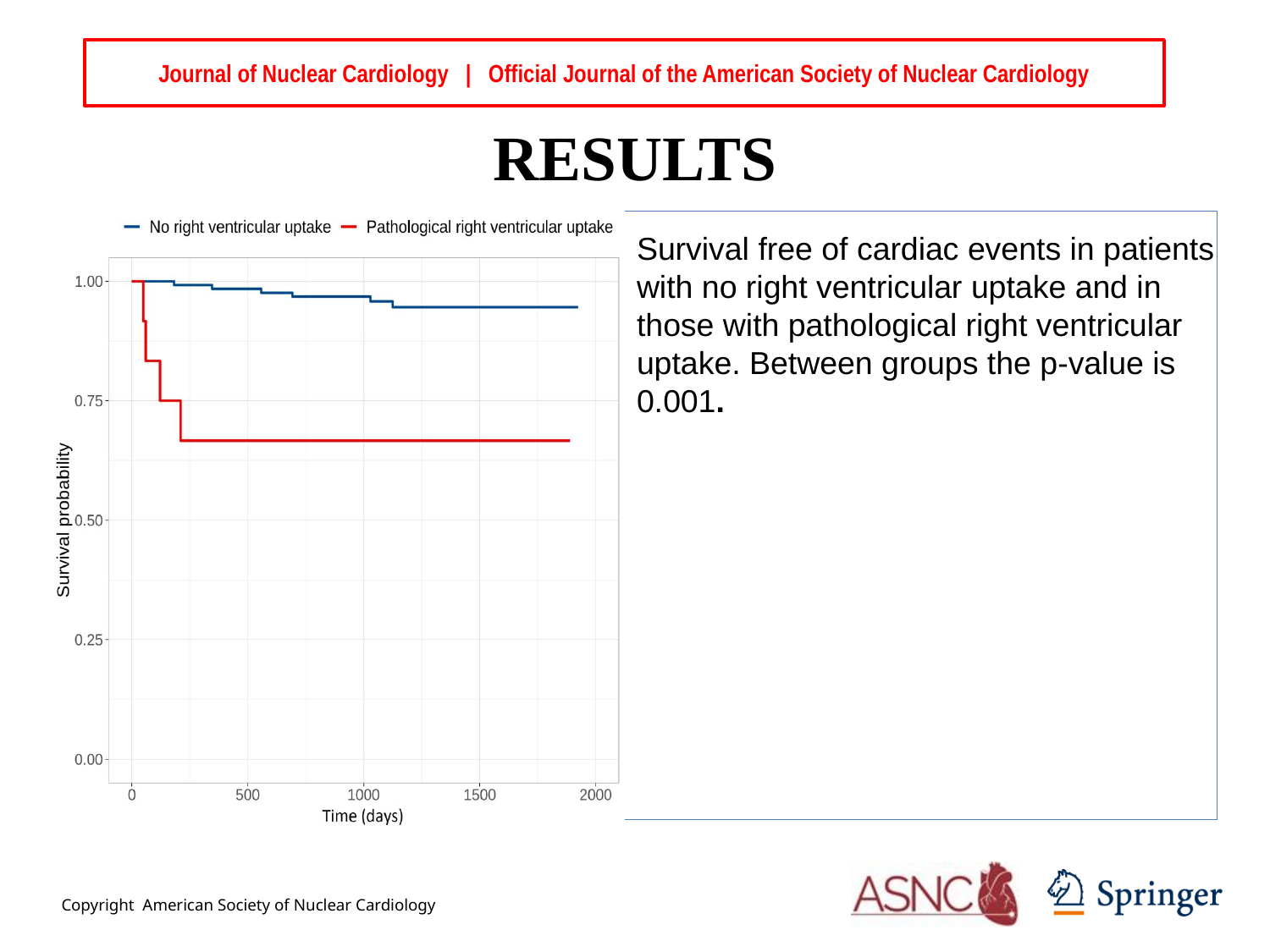

Journal of Nuclear Cardiology | Official Journal of the American Society of Nuclear Cardiology
# RESULTS
Survival free of cardiac events in patients with no right ventricular uptake and in those with pathological right ventricular uptake. Between groups the p-value is 0.001.
Copyright American Society of Nuclear Cardiology

## Slide 6
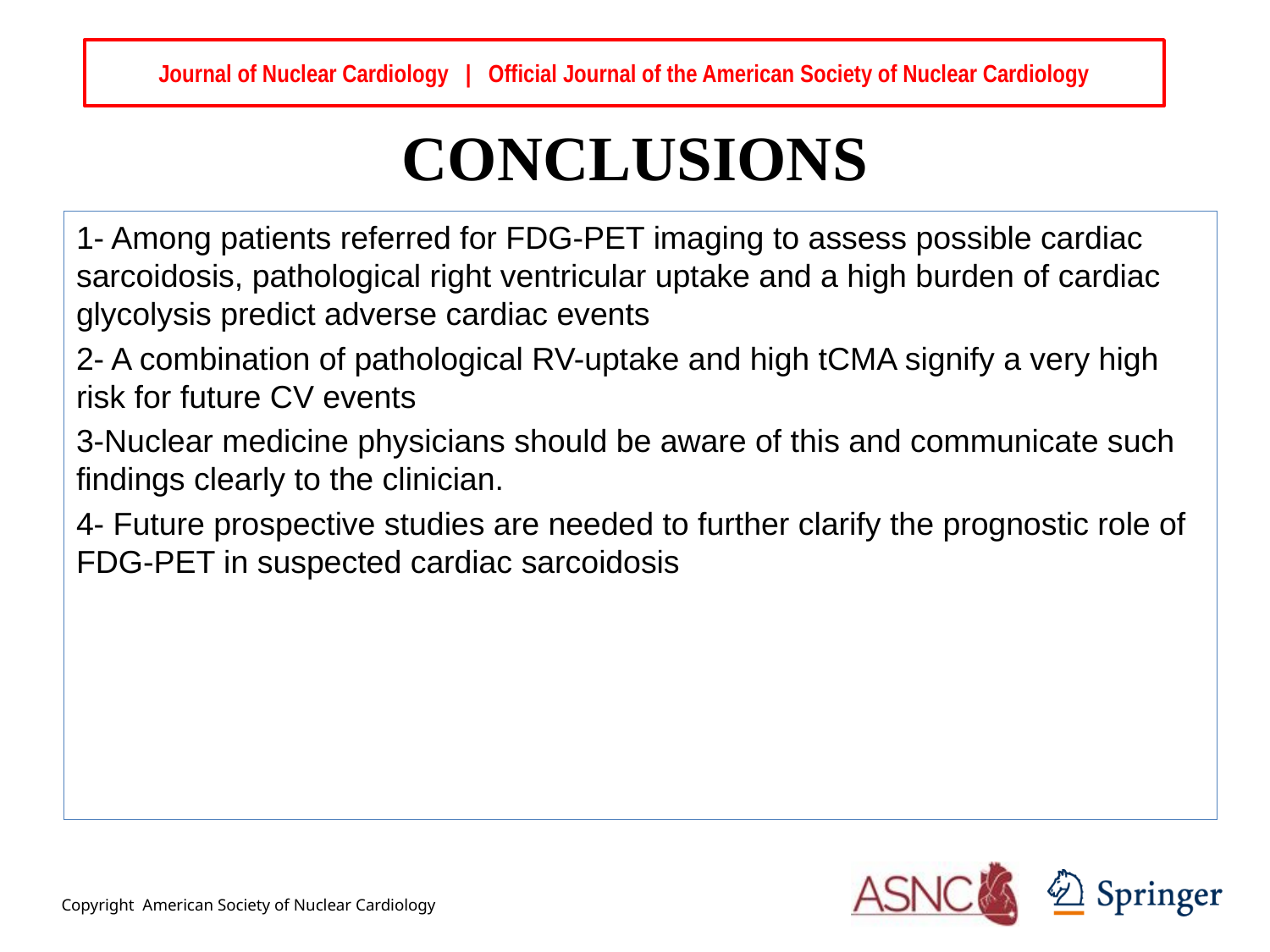

Journal of Nuclear Cardiology | Official Journal of the American Society of Nuclear Cardiology
# CONCLUSIONS
1- Among patients referred for FDG-PET imaging to assess possible cardiac sarcoidosis, pathological right ventricular uptake and a high burden of cardiac glycolysis predict adverse cardiac events
2- A combination of pathological RV-uptake and high tCMA signify a very high risk for future CV events
3-Nuclear medicine physicians should be aware of this and communicate such findings clearly to the clinician.
4- Future prospective studies are needed to further clarify the prognostic role of FDG-PET in suspected cardiac sarcoidosis
Copyright American Society of Nuclear Cardiology
